# Simulated Game-Based Ice Hockey Match Design (Scrimmage) Elicits Greater Intensity in External Load Parameters Compared With Official Matches

**DOI:** 10.3389/fspor.2022.822127

**Published:** 2022-02-17

**Authors:** Per Thomas Byrkjedal, Live Steinnes Luteberget, Thomas Bjørnsen, Andreas Ivarsson, Matt Spencer

**Affiliations:** ^1^Department of Sport Science and Physical Education, University of Agder, Kristiansand, Norway; ^2^Department of Physical Performance, Norwegian School of Sport Sciences, Oslo, Norway; ^3^Center of Research on Welfare, Health and Sport, Halmstad University, Halmstad, Sweden

**Keywords:** Local Positioning System (LPS), game-based training, team sports, inertial measurements units (IMU), athlete monitoring

## Abstract

**Objective:**

A limited number of studies have explored the external load experienced in indoor sports such as ice hockey, and few the link between training and match performance. As a paucity exists within this topic, this study explored whether a simulated match design (i.e., scrimmage) could be representative of official match demands and elicit similar external loads as in official matches in a group of elite youth male ice hockey players.

**Methods:**

A total of 26 players were monitored during eight official and four simulation matches using a Local Positioning System. Total distance, max velocity, slow (0–10.9 km/h), moderate (11–16.9 km/h), high (17.0–23.9 km/h), and sprint (>24 km/h) speed skating distance, distance per min, PlayerLoad^TM^, PlayerLoad^TM^ per min, high-intensity events (HIEs) (>2.5 m/s^−2^), acceleration (ACCs), decelerations (DECs), and change of directions (CODs) were extracted from the tracking devices. A two-level regression analysis was conducted to compare the difference between match types when controlling for time on ice, match day, and position.

**Results:**

Between match-type results showed a credible difference in all variables except max velocity and ACCs. Distance per min was 27.3% higher during simulation matches and was explained by a 21.3, 24.1, and 14.8% higher distance in sprint-, high-, and moderate speed skating distance, while slow speed-skating distance was 49.2% lower and total distance only trivially different from official to simulation matches. Total PlayerLoad^TM^ was 11.2% lower, while PlayerLoad^TM^ per min was 8.5% higher during simulation matches. HIEs, CODs, and DECs were 10.0, 11.9, and 22.3% higher during simulation matches.

**Conclusion:**

The simulated match design is related to official match demands with comparable match-time, playing time, number of shifts, and shift duration. However, simulation matches provoked a higher external load output compared with official matches, possibly explained by a more continuous movement design. A game-based simulation match design can therefore be utilized when match-related actions at high intensity are warranted.

## Introduction

Quantification of the external load has allowed for more extensive monitoring of training practices and can be used as an objective tool to optimize training and prepare for competitive performance. Match demands can be quantified at a team-, position- or individual-specific level. Recent studies of match-demands in ice hockey players have shown that players typically cover 50% of total distance in high-velocity zones (>17.0 km/h) (Lignell et al., 2018; Douglas and Kennedy, 2019). This is in contrast to running-based field sports, where most of the distance covered is in moderate-to-low-intensity zones, and only 10–20% in high-intensity zones (Bradley and Ade, 2018; Johnston et al., 2018; Kapteijns et al., 2021). The intermittent style of play with short, high-intensity shifts being performed throughout the match may be a reason for this. A typical shift lasts 45–60 s and involves 5–7 high-intensity actions, followed by 2–5 mins of rest on the bench before the subsequent shift (Brocherie et al., 2018; Vigh-Larsen et al., 2020; Wagner et al., 2021).

Further analysis of ice hockey match demands has revealed significant differences in intensity distribution between positions, periods, and odd-man situations (Douglas and Kennedy, 2019). Typically, forwards cover more distance in high-intensity zones (>17 km/h) compared with defensive players (Lignell et al., 2018; Douglas and Kennedy, 2019; Allard et al., 2020) and both total distance and intensity have been shown to decline from 1st to 3rd period (Brocherie et al., 2018; Lignell et al., 2018; Douglas and Kennedy, 2019; Douglas et al., 2019a; Allard et al., 2020). Interestingly, one study by Douglas et al. (2019b) compared the external load difference between training and matches in a group of elite female players. They found a clear mismatch in both intensity and volume between training and matches, which may partly explain the decline in match intensity across periods, as training seemed to be performed with an insufficient intensity level. This is supported by the findings of Spiering et al. (2003) as they demonstrated significantly lower heart rate distribution in training compared with matches (76 ± 3 vs. 90 ± 2% of HR_max_). Furthermore, Allard et al. (2020) recommended more match-like intensity during training drills, after assessing intensity distribution across a whole season.

Game-based training drills and scrimmage have been adopted in several sports to mimic specific match demands to address the experienced match complexity during training (Aguiar et al., 2012; Luteberget et al., 2018; Vazquez-Guerrero et al., 2021). However, there seems to be a lack of research on this topic within ice hockey. Lachaume et al. (2017) investigated energy expenditure during different drills by using heart rate, however, they only compared the drills between each other and not to match intensity. Other studies have used repeated-sprint protocols in an attempt to simulate ice hockey match performance in order to assess the physiological impact (Palmer et al., 2017a,b; Steeves and Campagna, 2019). To the authors knowledge, only Vigh-Larsen et al. (2020) have applied an actual game-based design in their attempt to replicate physical match demands. In a standardized simulation match, each player performed eight 1-min shifts per period and thereby total match time of ~24 min. Even though total distance was somewhat higher than previously reported, an intensity distribution similar to Douglas and Kennedy (2019), where ~50% of total distance was covered in high-intensity zones, was evident. Notably, the previously reported intensity decline toward the end of the match, was not evident in the simulation match. As previous studies have shown that there appears to be a mismatch between training- and match intensity, and a paucity exists in the ice hockey literature regarding the match transferability of game-based training drills, we wanted to explore if a simulated match design could be representative of match-like intensities. Furthermore, as there have been few studies examining the external load demands of modern-day ice hockey, we wanted to add further insights to the actual in season on-ice physical performance of male ice hockey players. Thus, the aim of this study was to assess whether a simulated match design could be representative of official match demands and elicit similar external loads as in official matches.

## Methods

In this study, we investigated the on-ice external load of official ice hockey matches and compared it to simulation matches. The study was conducted from September to December 2020 and included eight official matches and four simulation matches.

### Subjects

A group of 48 male players from a U21- and U18 team volunteered to participate in this study. Players had to wear an Local Positioning System (LPS) unit and participate in a minimum of four official matches, with a minimum of 5 min time on ice per match and/or all the four simulation matches to be included in the study. In total, 25 players were involved in official matches, where nine of the 25 players did not fulfill the inclusion criteria for official matches. Simulation matches initially included a squad of 34 players. Eleven players were excluded because of an insufficient number of LPS devices. Eight players were excluded due to promotion to the senior team (*n* = 1), injury (*n* = 3), and not participating in all matches (*n* = 4). Thus, 16 players (age: 18.7 ± 0.9 years, height 179.3 ± 4.8 cm, body mass 73.6 ± 4.9 kg) are included from the official matches and 15 players (age: 17.9 ± 1.1 years, height: 179.7 ± 6.4 cm, body mass: 72.3 ± 7.2 kg) are included from the simulation matches. Only U21 players are included from the official matches, while simulation matches included players from both teams (*n* = 8 U21 players and *n* = 7 U18 players). Of all the included players, six U21 players participated in both official and simulation matches. Thus, a total sample of 25 players (*n* = 9 DEF, *n* = 16 FWD) is included in this study. Written informed consent was obtained from all the players before initiating the study. The study was performed according to the ethical standards established by the Helsinki declaration of 1975 and was approved by the local ethical committee at the University of Agder, Kristiansand, Norway.

### Design

All the matches were played at the same arena, housing a North American-sized ice-rink (~60.96 × 25.9 m). An LPS system (Catapult Clearsky T6, Catapult Sports, Australia) was installed in the arena. A total of 20 anchor nodes were mounted ~20 m above the ice-surface. The system was spatially calibrated using a tachymeter (Leica Builder 509 Total Station; Leica Geosystems AG Switzerland), as recommended by the manufacturer. For both simulation and official matches, each player was equipped with an LPS unit (Catapult Clearsky T6, Catapult Sports, Australia: firmware version 5.6). The LPS unit was located between the scapulae in a specialized sewn vest supplied from the manufacturer.

#### Official Matches

Data from official matches were obtained from eight home matches played between September to November. Apart from wearing the LPS-unit, the study did not intervene with any aspect of the normal match or match preparation for players. The data collection was monitored in real-time using Catapult Openfield (Catapult Sports, Australia) Software (version 1.17.2). Interchanges were manually tracked using the software to ensure that only on-ice time was included in the analyses.

#### Simulation Matches

The simulation matches were standardized by modifying official match regulations, comparable to the simulation match in the study by Vigh-Larsen et al. (2020) Such gameplay replication may also be referred to as scrimmage (Vazquez-Guerrero et al., 2021). Accordingly, the simulated matches consisted of 3 × 20 min periods, interspersed by 18 min of recovery, in compliance with official match regulations. However, to standardize playing time, periods consisted of 20 min continuous play, without interference or stoppages. Changes of lines were performed every 1 min with a whistle-signal from the coach. At the whistle, all players on the ice performed a rapid change before the new line-up could enter the ice and immediately continue the play, resulting in a 1:2 work to rest ratio. Thus, the playing time for each player was ~20 min per match. In total, each match lasted 1 h and 36 min, including intermissions. To avoid odd-man situations, no penalties were given. However, to standardize play to normal match regulations and avoid reckless play, fictive penalties were used: for every second minor foul committed by the same team (i.e., 2-min penalty fouls), a goal was awarded to the opposition. If an offside or icing-situation occurred, the defensive team would gain possession of the puck. When a goal was scored, the play was immediately restarted by the goalkeeper taking out the puck from the net.

The players were allocated by the team coaches into two separate teams to give a balanced opposition for the simulation matches. Each team consisted of 15 players making three line-ups, where the best players (1st and 2nd line of each team) wore an LPS unit. The four simulation matches were arranged within a two-week period and played at the same time of day (±2.5 h). Players were verbally coached during every match and were given a tactical and motivational talk between periods, as in the official match situations. The data collection was monitored in real-time, and interchanges were manually tracked in the same way as in official matches.

### Data Processing

Total distance, distance per min, distance in speed skating zones, max velocity (max vel), PlayerLoad™, PlayerLoad™ per min, accelerations (ACCs), decelerations (DECs), and change of directions (CODs) were extracted from the Openfield software. Speed skating zones thresholds were chosen in accordance with previous research (Douglas and Kennedy, 2019; Vigh-Larsen et al., 2020) divided into slow (0–10.9 km/h), moderate (11.0–16.9 km/h), high (17.0–23.9 km/h), and sprinting (> 24 km/h) speed skating. PlayerLoad^TM^, high-intensity events (HIEs), ACCs, DECs, and CODs were applied as previously reported by Luteberget and Spencer (Luteberget and Spencer, 2017). Briefly, PlayerLoad^TM^ is calculated by taking the square root of the sum of the squared instantaneous rate of change in acceleration in each of the 3 vectors (x, y, and z axes), divided by 100 (Boyd et al., 2011). PlayerLoad^TM^ per min is calculated by dividing PlayerLoad^TM^ by the duration of the activity. ACCs, DECs, and CODs is a summary of identified movements in the respective direction with an intensity > 2.5 m/s^−2^. The sum of ACCs, DECs, and CODs was displayed as HIE. The data were edited postmatch to remove time between periods and time on the bench (i.e., only time on ice was included in the analysis). Data were extracted from the manufactures software and organized in Microsoft Excel (Microsoft Corp, Redmond, WA, USA).

### Statistics

Descriptive results were calculated using Microsoft Excel. Effect size of < 0.2, 0.2 to 0.6, 0.6 to 1.2, 1.2 to 2.0, and > 2.0 were considered trivial, small, moderate, large, and very large, respectively (Hopkins et al., 2009). Data are presented as mean ± SD and 95% CI. A 95% CI without crossing zero was decided to indicate a statistically significant result.

Bayesian 2-level regression analyses were performed in MPlus software (Muthén & Muthén. Los Angeles, CA, version 8.4) to assess potential associations between match type and the dependent variables. In the 2-level regression analyses, aimed to analyze data that contains an inherent hierarchical structure, every match data point for each player was set at level 1 (within a person) and is nested within individuals on level 2. Covariates can then be regressed on both levels. The advantages of using the Bayesian approach, in comparison to the more traditional frequentist approach are, for example, the increased likelihood of producing reliable estimates in small samples and the less restrictive distributional assumptions [for a more comprehensive comparison between the two approaches see, for example, Stenling et al. (2015)].

We applied the potential scale reduction factor to assess the model convergence and < 1.1 was considered as evidence of convergence (Kaplan and Depaoli, 2012). Model convergence was assessed using both statistical criteria and visual inspection of trace plots to ensure that multiple chains converged toward a similar target distribution (McArdle and Nesselroade, 2014). Bayesian models were implemented using Markov chain Monte Carlo simulation procedures with a Gibbs sampler and specified a fixed number of 150.000 iterations (the first half is used as the burn-in phase, which is the default in Mplus).

We used the posterior predictive *p* (PPp) value and the 95% CI to assess model fit. A well-fitting model should have a PP*p*-value around 0.50 in combination with asymmetric 95% CI centering on zero. We also inspected the root mean square error of approximation, comparative fit index, and Tucker Lewis Index to determine the models fit to data. For each parameter, a credibility interval was estimated. If the 95% credibility interval does not include zero, the null hypothesis was rejected as improbable, and the parameter estimate is considered credible (Zyphur and Oswald, 2015). For all parameters default, priors in Mplus were used (Muthén and Asparouhov, 2012).

Based on the findings in the previous studies, we included several covariates potentially influencing match physical performance (Brocherie et al., 2018; Perez et al., 2020). Match type, match day (only official matches), and playing time (time on ice) was used as predictor variables within level (level 1). Playing position was used as a predictor variable between level (level 2) and defensive/forward players were coded “0/1”, respectively. Interpretation of results from the regression analysis should be done by comparing beta-coefficients (e.g., a beta-coefficient value of −0.6 is stronger than −0.5).

## Results

A summary of the included external load variables during official and simulation matches can be found in Table 1. Players appeared in 6.8 ± 1.5 official matches and 4.0 ± 0.0 simulation matches. Average time on ice for the respective match types was 26:28 ± 09:45 min (range: 05:02–44:40) during official matches and 21:00 ± 00:14 min (range 20:13–21:22) during simulation matches. On average, players had 22.9 ± 7.4 and 20.0 ± 0.0 shifts per match, with the average time on ice per shift being 67.7 ± 8.7 s and 63.0 ± 0.7 s for official and simulation matches, respectively. When excluding intermissions, official and simulation matches lasted 88.3 ± 7.2 and 60.0 ± 0.0 min, respectively.

**Table 1:** Match data from the included variables during official and simulation matches.

	**Match Type**			
**Variable**	**Official (*n* = 109)**	**Simulation (*n* = 60)**	**ES**	**95% CI**
Total distance	4,894 ± 1,731	5,015 ± 502	0.09	−0.23/0.40
Max Vel	8.50 ± 0.52	8.39 ± 0.54	−0.22	−0.53/0.10
Slow speed skating	1,228 ± 486	624 ± 166	−1.49	−1.84/−1.14
Moderate speed skating	1,547 ± 587	1,775 ± 267	0.46	0.14/0.77
High speed skating	1,744 ± 683	2,164 ± 628	0.63	0.31/0.95
Sprint speed skating	365 ± 228	442.4 ± 285	0.31	−0.01/0.63
Distance per min	188 ± 18	239 ± 24	2.51	2.09/2.92
Total PlayerLoad^TM^	161.3 ± 59.8	143.3 ± 27.7	−0.35	−0.67/0.04
PlayerLoad^TM^ per min	6.3 ± 1.2	6.8 ± 1.3	0.42	0.10/0.74
HIE	237.8 ± 79.3	261.7 ± 63.7	0.32	0.00/0.64
ACC	15.6 ± 10.1	9.3 ± 4.8	−0.73	−1.05/−0.41
DEC	35.4 ± 15.2	43.3 ± 15.0	0.52	0.20/0.84
COD	186.7 ± 65.1	209.2 ± 56.0	0.36	0.04/0.68

*Mean ± SD*.

*HIE, high-intensity event; ACC, acceleration; DEC, deceleration; COD, change of direction*.

**Figure 1:**
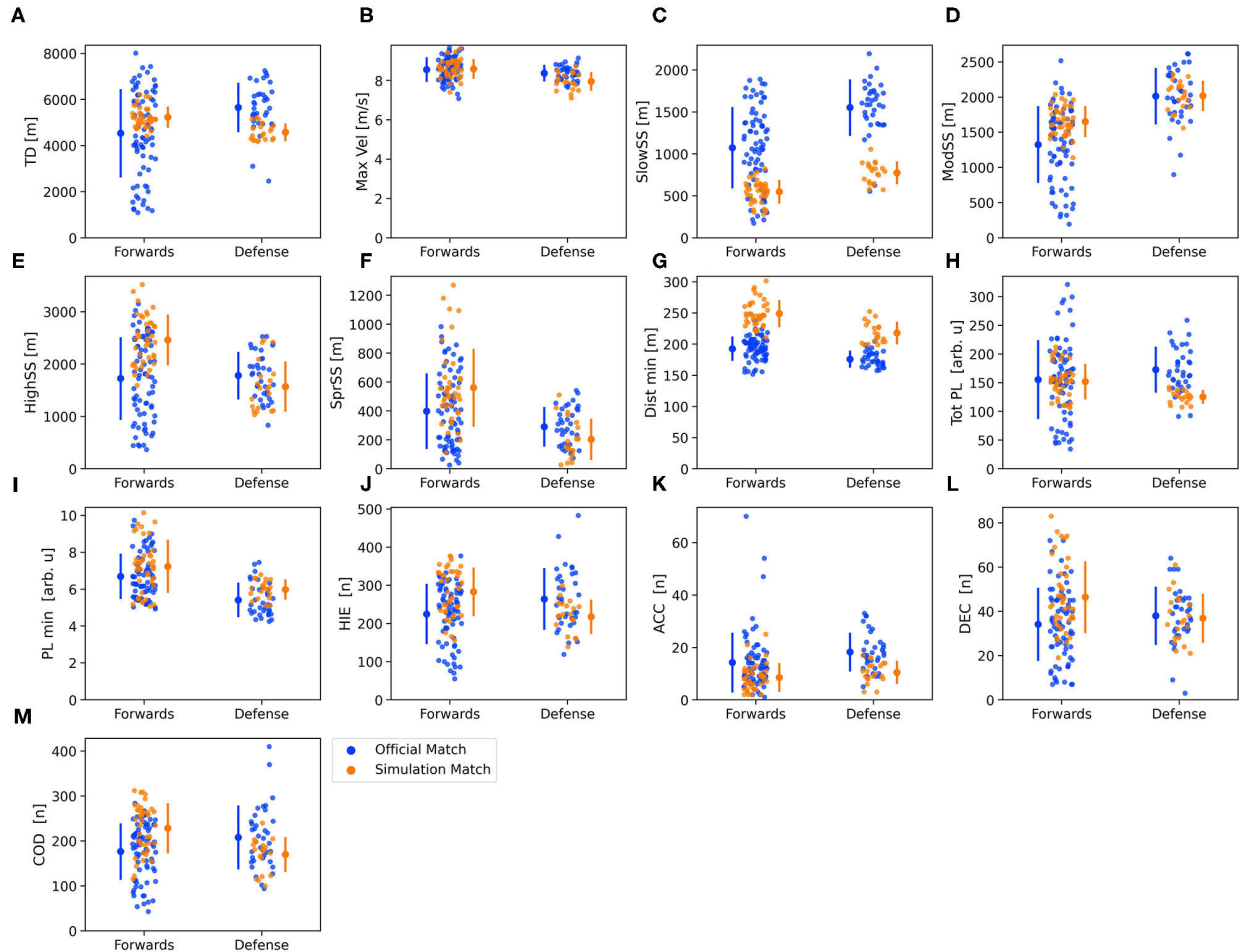
Positional differences between official- (blue) and simulation (yellow) matches for the included locomotive and inertial sensor variables. Data are presented as mean ± SD and individual plots for each variable. **(A)** Total distance, **(B)** Max vel, **(C)** Slow speed skating, **(D)** Moderate speed skating, **(E)** High speed skating, **(F** Sprint speed skating, **(G)** Distance per min, **(H)** Total PlayerLoad^TM^, **(I)** Total PlayerLoad^TM^ per min, **(J)** High intensity events, **(K)** Acceleration, **(L)** Deceleration, and **(M)** Change of direction. TD, total distance; SS, speed skating; Dist min, distance per min; PL, PlayerLoad^TM^; PL min, PlayerLoad^TM^ per min; HIE, high-intensity event; ACC, acceleration; DEC, deceleration; COD, change of direction.

The model fit from the 2-level regression had an acceptable PP*p*-value for all variables (range 0.467 to 0.474) and results can be found in Table 2. Match type had a credible impact on all dependent variables, except max vel and ACCs when controlling for match day and time on ice. Total distance was strongly related to time on ice, and a trivial difference can be observed between match types (Tables 1, 2). When comparing the impact of match type on the included variables, a stronger impact was evident for high speed skating, distance per min, and sprint speed skating, which was 24.1, 27.3, and 21.3% higher during simulation matches. Furthermore, a weaker impact was evident for slow- and moderate speed skating distance which, compared to official matches, were 49.2% lower and 14.8% higher in simulation matches. For inertial measurement unit data, match type seems to have a stronger impact on CODs, HIEs, and DECs (11.9, 10.0, and 22.3% higher during simulation matches), compared to total PlayerLoad^TM^ and PlayerLoad^TM^ per min (11.2% lower and 8.5 higher during simulation matches, respectively). ACCs had a 40.6%, but noncredible, lower value for official- in comparison to simulation matches. The position had a credible between-level influence on all variables except ACCs and CODs. Data for each position is shown in Figure 1.

**Table 2:**
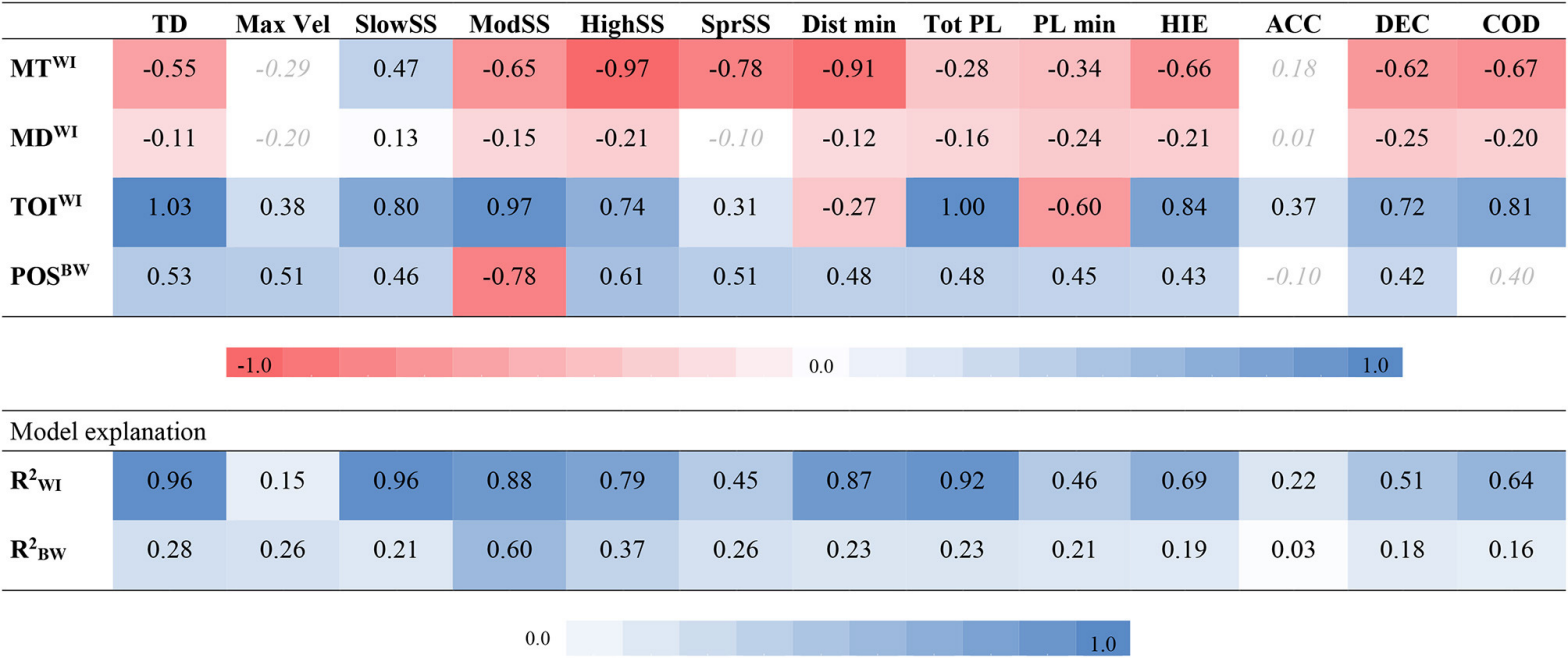
Results from the 2-level regression analysis.

*Significant beta-coefficient- (range: −1.0 to 1.0) and R2 (range: 0.0 to 1.0) strength is displayed by graded color backgrounds. Gray italic font numbers on white background are indicating not credible estimates*.

*MT, match type; MD, match day; TOI, time on ice; POS, position; WI, with-in level; BW, between-level; TD, total distance; SS, speed skating; Dist min, distance per min; PL, PlayerLoad^TM^; PL min, PlayerLoad^TM^ per min; HIE, high-intensity event; ACC, acceleration; DEC, deceleration; COD, change of direction*.

## Discussion

The main aim of this study was to assess if a simulated match design could be representative of official match demands and elicit similar external loads as in the official matches. We are, to the knowledge of the authors, the first to compare external load measurements from a simulated match design to external load from official matches in ice hockey. With similar match time, playing time, a number of shifts, and shift duration, the simulated match design provides an environment comparable to official matches. However, our results show differences in on-ice physical performance, with players eliciting a higher intensity in simulation matches. The results of this study provide practitioners with a game-based training drill applying a matching design that could be used in training situations where match-specific tasks at high intensity are warranted.

We observed a very large difference in distance per min, which can be seen in relation to the small-to-large difference in distance covered in speed skating zones. While slow speed skating was lower, moderate-, high-, and sprinting speed skating distance were all higher in simulation matches, compared to official matches. With a similar total distance covered between match types, our results, therefore, suggest that during simulation matches, a portion of distance covered in the low-intensity zone is replaced with more distance in higher speed zones. Naturally, a higher distance per min is undertaken in simulation matches because of the opportunity to continuously be on the move and chase the puck with opposition pressure. In contrast, the lower distance per min observed in official matches is explained by the inclusion of low activity data during stoppage time. Similar findings are, for example, also shown in basketball (Svilar et al., 2019). However, when comparing effective playing time from simulation matches to time on ice in official matches, the relative playing time is ~30–35% of total match time in both match conditions. Thus, our official match data, including playing time, number of shifts, and shift duration, are comparable to previously reported observations from official matches (Brocherie et al., 2018; Lignell et al., 2018; Douglas and Kennedy, 2019). Our simulation match design, therefore, seems appropriate when coaches wish to address high-intensity actions, avoiding low-intensity actions, while at the same time keeping a game-based design in training situations.

When comparing our results from the simulation match to the results from the simulation match of Vigh Larsen et al., the intensity distribution between the two studies is similar, with the exception of a higher distance in the slow intensity skating sone in the study of Vigh-Larsen et al. (2020) This may be due to inclusion of stoppage in play. In contrast, the same authors displayed a markedly higher total distance compared with our study (Vigh-Larsen et al., 2020). However, if comparing total distance related to playing time, distance per min is quite comparable. The observed difference in total distance might be due to their design, applying 24 min periods compared to our 20 min periods, allowing each player to complete ~4 min higher match time, and thus a higher total distance. Notably, our design, using less time and no stoppages, while eliciting similar high-intensity distance and distance per min as Vigh-Larsen et al. (2020), therefore, seems to be a sufficient method to time-effectively simulate game play in training situations.

Our results show a small difference in total PlayerLoad^TM^ and PlayerLoad^TM^ per min. While total PlayerLoad^TM^ was lower, PlayerLoad^TM^ per min was higher during simulation matches. This is likely explained by the premises of match play, as an increased relative intensity has also been shown during no stop match play compared with the official rule match play in basketball (Svilar et al., 2019). As PlayerLoad^TM^ measure is a measure of the sum of forces (x, y, and z axes) generated through the accelerometer, the lack of start/stops that typically occurs when stopping the play or dropping the puck in official matches, might be the reason for this decline in total PlayerLoad^TM^. Similar to distance per min, the more continuous movement and only obtaining data while the puck is in play, seems like the logical explanation for the small increase in PlayerLoad^TM^ per min during simulation matches. When comparing PlayerLoad™ from matches, our results are lower than Neeld et al. (2021) reported in collegiate level male players (total PlayerLoad^TM^ 220–234 [DEF-FWD]) and what Douglas et al. (2019b) reported in two other studies in elite female players; 230–239 [DEF-FWD] and Douglas et al. (2020); 228–246, [DEF-FWD]). This difference might be attributed to the methodological differences (inclusion of data), however, playing level and athlete caliber (Perez et al., 2020) could also contribute to the observed differences. Contrastingly, elite and subelite female players have also displayed a total PlayerLoad^TM^ comparable to our results [total PlayerLoad^TM^ 153–159 [DEF-FWD] Douglas et al. (2019a); and 160–183 [FWD-DEF] Douglas et al. (2020)]. PlayerLoad^TM^ per min has been reported in three of the studies (Douglas et al., 2019b, 2020; Neeld et al., 2021), however, none of them excluded time on the bench, which makes it challenging to compare this metric to our results. Similar to our study, however solely using the inertial movement unit-device within the Clearsky T6-unit, Allard et al. (2020) applied on ice load to quantify external load in a group of fifty male American Hockey League players over an entire season. On ice load was intended to be a more representative and precise measure of PlayerLoad^TM^ in ice hockey, removing all low ACCs (<0.3 m/s^−2^) that typically occur (i.e., time on the bench, substitutions, coasting, gliding, standing, and resting) but at the same time comparable (Pearson correlation: *R*^2^ = 0.98). Assuming that PlayerLoad^TM^ can be compared to on ice load, the match results from Allard et al. (2020) were comparable to total PlayerLoad^TM^ in our and previous studies (total on ice load 139–151 [DEF-FWD]). However, on ice load per min was 11.8–13.8 (DEF-FWD), which is markedly higher than our results. No other studies have reported comparable measures for HIEs, DECs, or CODs in ice hockey, however, the higher number of observed actions from official to simulation matches strengthens the assumptions of an overall higher intensity during simulation matches.

ACCs was not different between match types. Increased focus on acceleration has been highlighted in other sports due to the metabolic demands and power output needed to increase speed compared to maintaining a constant high speed (Cardinale and Varley, 2017), and it is natural to think that this measure is highly transferable to ice hockey. In contrast to running-based sports such as soccer, handball, rugby, American/Australian football, basketball, etc., it is relatively easy to remain at high speed because of the low friction on the ice. Therefore, the importance of acceleration in addition to locomotive speed distance measures should be highlighted in future studies applying LPS systems. Furthermore, even though a continuous play design has been applied in other sports, the consequence and the potential interference this has on acceleration-derived data, should be further investigated within ice hockey.

Douglas and Kennedy (2019) only included effective playing time when assessing the performance of the male Canadian U20 team during international matches. Interestingly, slow speed skating distance seems comparable, while moderate- and high speed skating distance is higher during our simulation matches. Contrastingly, sprint skating distance is higher during the international matches compared to our simulation matches. Athlete caliber, in addition to variations in methodology, seems like a possible explanation for this observed difference. The methodology is an important debate, as our match data during both match types shows comparable or higher distances covered in high-intensity zones (>17 km/h) compared to professional National Hockey League players (Lignell et al., 2018). Even though our official match results included distance covered while the puck was out of play, its unlikely to think that this is the reason for the superior distance covered in the high-intensity distance zones. A suggested explanation is an increased sensitivity when applying an LPS system compared with a semiautomated tracking system.

There are some limitations that should be considered. At first, in contrast to other studies on ice hockey, our design allows players to accumulate time on the ice when the puck is out of play (in official matches). This approach has also been used in other team sports (Luteberget and Spencer, 2017) and takes into account all load when players are on-ice. However, this approach will allow players to reach higher playing time and affect parameters such as distance- and PlayerLoad^TM^ per min. In our study, we find the percentage on-ice time (26:28 ± 9:45: 19–41% of total time) to be in line with previous literature reporting 15–25 min of effective playing time (25–42% of total match time). Therefore, we think this approach is appropriate, although the difference from other studies could affect the direct comparability. Second, our official match data only included the home team. Furthermore, the within-level performance seems to be negatively impacted by the second matchday during consecutive matches during official matches, as this is a significant predictor in all variables except max vel, sprint speed skating, and ACCs. However, even though the impact is weak (beta-coefficient range −0.24 to .13), and this schedule is typical for this team, playing against the same opposition over two back-to-back match days may differ from other match schedules and complicate data comparison. Furthermore, the match score is an important factor as players are likely to improve their efforts if there is an even score or when chasing an equalizer, compared with leading 10–3 the last minutes of the game, which was the case in one of the included matches (Brocherie et al., 2018). This will further influence the tactical decisions, such as player rotations, playing time, etc. Caution should be taken when trying to generalize the findings as many factors contribute to the overall physical performance (overall fitness level, technical skills, athlete caliber, tactical strategies, etc.). Furthermore, the study was conducted in the middle of a pandemic. Lack of gym facilities during preseason, no ice during the summer period, psychological factors (stress, anxiety), etc., are all the factors influencing overall performance in this time period. However, this unique situation also gave a special opportunity (e.g., if the players performed well, a promotion to the senior team was more likely to occur due to injuries, quarantines, etc.). Therefore, the players had to remain fit and fully motivated for the entire period. At last, different calculations of acceleration load (i.e., PlayerLoad™, on ice load, Accel'Rate) used in the literature complicate comparison and interpretation of the results. Indeed, the PlayerLoad^TM^ calculations have recently been suggested to have limitations for estimating whole-body mechanical load (Hollville et al., 2021), and its relevance in ice hockey is not investigated. Implementation of Accel'Rate has been suggested as a more sensitive measure (Hollville et al., 2021) and has indeed been applied in ice hockey (Perez et al., 2020) and future studies should assess its relevance compared to the traditional use of PlayerLoad^TM^.

### Practical Applications

This study suggests that a game-based match design can be adopted when practitioners wish to address match-like performance with intensity- and high-speed skating distance (> 17 km/h) superior to official matches during training drills. Within the field of game-based training-drills in ice hockey, more research is needed to assess the external load and intensity when the drill design is manipulated (number of players, rink size, etc.) and how this is linked to external load during official matches. Some caution should be taken when interpreting the results. Even though a simulation match design is comparable to the official matches, there are large individual variations. For example, during the official matches players are exposed to as much as 44 min or as little as 5 min time on the ice. Accordingly, simulation matches, with a standardized time on the ice, will be an over or underrepresentation of actual match demands for some players. It is, however, a feasible way of eliciting the match-like intensity that has been lacking in the previous studies.

## Conclusion

This study shows that there is a difference in the external load parameters between official and simulation matches. Specifically, a higher distance per min and more distance covered in the moderate-, high-, and sprinting speed skating zones is observed in the simulation matches, compared with official matches. This difference seems to be associated with the continuous play design, allowing players to always be on the move and thus display a higher intensity during simulated matches. Our findings provide practitioners with a game-based match design that can be adopted during training situations when match-specific training is desired.

The authors declare that the research was conducted in the absence of any commercial or financial relationships that could be construed as a potential conflict of interest.

## Data Availability Statement

The raw data supporting the conclusions of this article will be made available by the authors, without undue reservation.

## Ethics Statement

The studies involving human participants were reviewed and approved by Fakultetets etiske komité (FEK) ved Faktultetet for helse- og idrettsvitenskap, Universitetet i Agder. The patients/participants provided their written informed consent to participate in this study. Written informed consent was obtained from the individual(s) for the publication of any potentially identifiable images or data included in this article.

## Author Contributions

PB, LL, TB, and MS contributed to the conceptions and design of the study. PB, LL, and TB executed the study and collected data. AI and PB performed the statistical analysis. PB wrote the first draft of the manuscript. All the authors contributed to manuscript revision, read, and approved the submitted version.

## Funding

After acceptance, the local institution (University of Agder) will cover expenses related to open access publication.

## Conflict of Interest

The authors declare that the research was conducted in the absence of any commercial or financial relationships that could be construed as a potential conflict of interest.

## Publisher's Note

All claims expressed in this article are solely those of the authors and do not necessarily represent those of their affiliated organizations, or those of the publisher, the editors and the reviewers. Any product that may be evaluated in this article, or claim that may be made by its manufacturer, is not guaranteed or endorsed by the publisher.
